# Unusual uterine metastasis of invasive ductal carcinoma: A case report

**DOI:** 10.4274/tjod.88964

**Published:** 2016-09-15

**Authors:** Tayfur Çift, Berna Aslan, Berk Bulut, Şennur İlvan

**Affiliations:** 1 Bursa Yüksek İhtisas Training and Research Hospital, Clinic of Gynecology and Obstetrics, Bursa, Turkey; 2 Kanuni Sultan Süleyman Training and Research Hospital, Clinic of Gynecology and Obstetrics, İstanbul, Turkey; 3 Okmeydanı Training and Research Hospital, Clinic of Gynecology and Obstetrics, İstanbul, Turkey; 4 İstanbul University Cerrahpaşa Faculty of Medicine, Department of Pathology, İstanbul, Turkey

**Keywords:** uterus, ductal carcinoma, metastasis

## Abstract

Metastatic carcinoma of the uterus usually originates from other genital sites. Extragenital metastases such as breast are rare. A woman aged 34 years with a history of breast cancer was referred to the gynecology outpatient clinic for routine follow-up. Diagnostic tests and gynecologic examination revealed a uterine mass, which was removed with laparotomy. The pathologic investigation revealed metastasis of invasive lobular breast cancer. Chemotherapy was given and the patient has been under follow-up for 3 years with normal imaging on comput-erized tomographic examination and positron-emission tomography-computerized tomographic. It should be kept in mind that patients with breast cancer who have received tamoxifen may develop primary endometrial cancers, and may also demonstrate uterine metastases. With successful treatment these patients can obtain dis-ease-free survival.

## INTRODUCTION

Breast cancer usually spreads to the lungs, bone, liver, brain, gastrointestinal (GI) tract, peritoneum or retroperitoneum. Extramammarial metastasis of breast cancer such as in the uterus is rarely seen^(1,2,3,4)^.

Invasive lobular carcinoma (ILC) differs from infiltrating ductal carcinoma (IDC) with respect to sites of metas-tasis. IDC metastases are frequently seen in the lung, bones, and liver. However, ILC’s metastatic sites are GI peritoneum and the retroperitoneum^(3,4)^.

ILC of the breast spreads to gynecologic organs more frequently than invasive ductal carcinoma^(2,3)^. Recurrence of breast cancer usually occurs during the first two years. The incidence of recurrence decreases over time; however, it never disappears completely^(5)^. In this report, we present a patient with recurrent metastatic invasive ductal breast carcinoma in the uterus 12 years after the initial diagnosis, with 3 years survival following successful treatment.

## CASE REPORT

A woman aged 34 years with a history of ductal carcinoma of the breast was referred to the gynecology outpatient clinic for routine follow-up. She had a history of left-sided total mastectomy and axillary lymph node dissection due to a malignant mass in her left breast 12 years ago. The histopathologic result was reported as invasive ductal carcinoma and the patient underwent 6 courses of taxotere, adriamycin, cyclophosphamide chemotherapy and 25 days of radiotherapy (a total dose of 5000 cGy) following the operation. Hormonotherapy with tamoxifen was given for five years owing to the positive (90%) estrogen and progesterone hormone receptors.

**Figure 1:**
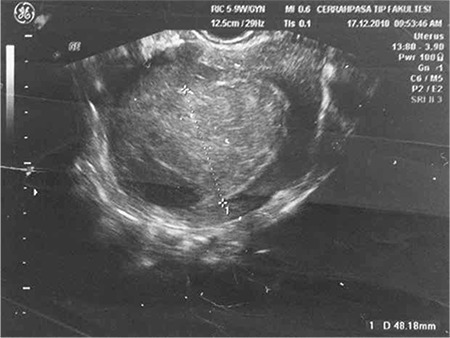
Ultrasound image of the uterine mass

The current diagnostic tests and gynecologic examination revealed a 4.8x3.2 cm lesion that caused diffuse thickening of the uterine wall (Figure 1). She was referred to the gynecologic oncology department for further evaluation. An endometrial biopsy was performed to eliminate any endometrial pathology caused by tamoxifen treatment, which was found negative.

**Figure 2:**
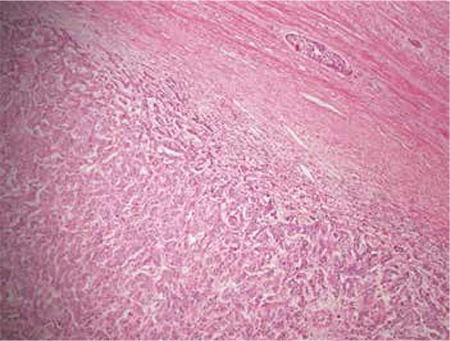
Pathologic examination of the excised material Pathologic examination. Pathologic microscopic examination demonstrating malignant epithelial tumor cells; (Hematoxylin-Eosin, x100)

The mass on the uterine wall was removed under laparotomy. The pathologic investigation revealed metastasis of invasive ductal carcinoma of the breast. Breast carcinoma metastases in the myometrium were confirmed histopathologically and immunohistochemically (Figure 2). The results of the pathologic investigation were consulted with the medical oncology department and additional chemotherapy with gemcitabine and capecitabine (1-8/28 days) was given to the patient. The chemotherapy was completed and the patient has been under follow-up for 3 years with normal imaging on computerized tomographic (CT) examination and positron-emission tomography-CT (PET/CT).

## DISCUSSION

Metastases to the female genital tract from extragenital cancers are rare. Breasts and the GI tract are the most common sites of the primary tumor. Ovaries are most frequently affected by metastases, which account for 75.8%, followed by the vagina (13.4%), uterine corpus (4.7%), cervix (3,4%), vulva (2%), and salpinx (0.7%)^(6)^. Uterine metastases usually occur secondary to local lymphatic spread due to ovarian involvement and thus isolated uterine metastases from the extragenital tumors are rare and probably hematogenous. The initial symptoms of uterine metastasis depend on the anatomic involvement site. If the infiltration affects only the myometrium, patients may often be asymptomatic, as seen in our case^(7)^.

To detect metastatic disease early, routine gynecologic follow-up examinations should always be performed, even in asymptomatic patients with breast cancer under tamoxifen therapy. Additionally, it is important to distinguish whether the uterine lesions are primary or metastatic because of the different treatment options. The uterus is an uncommon site for breast cancer metastasis. Nevertheless, most uterine metastases are found at autopsy^(2)^.

Tamoxifen has played an essential role in the treatment of hormone receptor-positive breast cancers. Five years of treatment with tamoxifen can reduce the risk of both recurrence and mortality due to breast cancer by approximately 30%^(7)^. However, tamoxifen exerts a partial agonistic effect on the endometrium. Therefore, treatment with tamoxifen increases the incidence of endometrial hyperplasia, polyps, and endometrial neoplasms^(8,9)^.

Precise diagnosis affects critical decision-making with regard to the treatment of the uterine cancer. A primary uterine tumor should be resected, whereas a metastatic uterine tumor should be treated with systemic therapy as the first choice^(10,11)^. In the present case, tamoxifen was used as an adjuvant hormone therapy with no additional surgical intervention.

Although there are few reports on the prognosis of patients with metastatic uterine tumors from breast cancer, most patients have been reported to have poor prognosis. However, given the limited number of reported cases, further studies are needed, along with a larger number of case reports to further our understanding of the prognosis of these cancers, and to determine the best course of treatment^(12)^. There has been no recurrence in our patient for the last 3 years and this is the longest disease-free survival report in such cases.

Any patient with abnormal imaging should be evaluated for vaginal, cervical, and uterine pathology. As this case aptly demonstrates, negative endometrial biopsy should not reduce the concern for pathology or alter the examination of a patient with abnormal imaging.

This case demonstrates the importance of maintaining a broad differential diagnosis in patients with abnormal imaging in the setting of a history of breast cancer and tamoxifen use. While performing ultrasound examination, like with this patient, focusing on endometrial thickness, endometrial polyps or suspicion of endometrial neoplasm may cause suspicious findings to be missed, except endometrium. In addition, endometrial biopsy alone is not sufficient for a diagnosis, and may be misleading. Evaluation of symptoms with imaging remains essential in identifying the underlying pathology.

In conclusion, it should be kept in mind that patients with breast cancer who have received tamoxifen may develop primary endometrial cancers, and may also demonstrate uterine metastases.
